# Machine learning-derived cycle length variability metrics predict spontaneously terminating ventricular tachycardia in implantable cardioverter defibrillator recipients

**DOI:** 10.1093/ehjdh/ztad064

**Published:** 2023-10-24

**Authors:** Arunashis Sau, Amar Ahmed, Jun Yu Chen, Libor Pastika, Ian Wright, Xinyang Li, Balvinder Handa, Norman Qureshi, Michael Koa-Wing, Daniel Keene, Louisa Malcolme-Lawes, Amanda Varnava, Nicholas W F Linton, Phang Boon Lim, David Lefroy, Prapa Kanagaratnam, Nicholas S Peters, Zachary Whinnett, Fu Siong Ng

**Affiliations:** National Heart and Lung Institute, Hammersmith Campus, Imperial College London, 72 Du Cane Road, W12 0HS, London, UK; Department of Cardiology, Hammersmith Hospital, Imperial College Healthcare NHS Trust, 72 Du Cane Road, W12 0HS, London, UK; National Heart and Lung Institute, Hammersmith Campus, Imperial College London, 72 Du Cane Road, W12 0HS, London, UK; National Heart and Lung Institute, Hammersmith Campus, Imperial College London, 72 Du Cane Road, W12 0HS, London, UK; National Heart and Lung Institute, Hammersmith Campus, Imperial College London, 72 Du Cane Road, W12 0HS, London, UK; Department of Cardiology, Hammersmith Hospital, Imperial College Healthcare NHS Trust, 72 Du Cane Road, W12 0HS, London, UK; National Heart and Lung Institute, Hammersmith Campus, Imperial College London, 72 Du Cane Road, W12 0HS, London, UK; National Heart and Lung Institute, Hammersmith Campus, Imperial College London, 72 Du Cane Road, W12 0HS, London, UK; Department of Cardiology, Hammersmith Hospital, Imperial College Healthcare NHS Trust, 72 Du Cane Road, W12 0HS, London, UK; National Heart and Lung Institute, Hammersmith Campus, Imperial College London, 72 Du Cane Road, W12 0HS, London, UK; Department of Cardiology, Hammersmith Hospital, Imperial College Healthcare NHS Trust, 72 Du Cane Road, W12 0HS, London, UK; National Heart and Lung Institute, Hammersmith Campus, Imperial College London, 72 Du Cane Road, W12 0HS, London, UK; Department of Cardiology, Hammersmith Hospital, Imperial College Healthcare NHS Trust, 72 Du Cane Road, W12 0HS, London, UK; National Heart and Lung Institute, Hammersmith Campus, Imperial College London, 72 Du Cane Road, W12 0HS, London, UK; Department of Cardiology, Hammersmith Hospital, Imperial College Healthcare NHS Trust, 72 Du Cane Road, W12 0HS, London, UK; National Heart and Lung Institute, Hammersmith Campus, Imperial College London, 72 Du Cane Road, W12 0HS, London, UK; Department of Cardiology, Hammersmith Hospital, Imperial College Healthcare NHS Trust, 72 Du Cane Road, W12 0HS, London, UK; National Heart and Lung Institute, Hammersmith Campus, Imperial College London, 72 Du Cane Road, W12 0HS, London, UK; Department of Cardiology, Hammersmith Hospital, Imperial College Healthcare NHS Trust, 72 Du Cane Road, W12 0HS, London, UK; National Heart and Lung Institute, Hammersmith Campus, Imperial College London, 72 Du Cane Road, W12 0HS, London, UK; Department of Cardiology, Hammersmith Hospital, Imperial College Healthcare NHS Trust, 72 Du Cane Road, W12 0HS, London, UK; National Heart and Lung Institute, Hammersmith Campus, Imperial College London, 72 Du Cane Road, W12 0HS, London, UK; Department of Cardiology, Hammersmith Hospital, Imperial College Healthcare NHS Trust, 72 Du Cane Road, W12 0HS, London, UK; National Heart and Lung Institute, Hammersmith Campus, Imperial College London, 72 Du Cane Road, W12 0HS, London, UK; Department of Cardiology, Hammersmith Hospital, Imperial College Healthcare NHS Trust, 72 Du Cane Road, W12 0HS, London, UK; National Heart and Lung Institute, Hammersmith Campus, Imperial College London, 72 Du Cane Road, W12 0HS, London, UK; Department of Cardiology, Hammersmith Hospital, Imperial College Healthcare NHS Trust, 72 Du Cane Road, W12 0HS, London, UK; National Heart and Lung Institute, Hammersmith Campus, Imperial College London, 72 Du Cane Road, W12 0HS, London, UK; Department of Cardiology, Hammersmith Hospital, Imperial College Healthcare NHS Trust, 72 Du Cane Road, W12 0HS, London, UK; National Heart and Lung Institute, Hammersmith Campus, Imperial College London, 72 Du Cane Road, W12 0HS, London, UK; Department of Cardiology, Hammersmith Hospital, Imperial College Healthcare NHS Trust, 72 Du Cane Road, W12 0HS, London, UK; National Heart and Lung Institute, Hammersmith Campus, Imperial College London, 72 Du Cane Road, W12 0HS, London, UK; Department of Cardiology, Hammersmith Hospital, Imperial College Healthcare NHS Trust, 72 Du Cane Road, W12 0HS, London, UK; Department of Cardiology, Chelsea and Westminster Hospital NHS Foundation Trust, 369 Fulham Road, SW10 9NH, London, UK

**Keywords:** Machine learning, Ventricular tachycardia, Cycle length, Implantable cardioverter defibrillator

## Abstract

**Aims:**

Implantable cardioverter defibrillator (ICD) therapies have been associated with increased mortality and should be minimized when safe to do so. We hypothesized that machine learning-derived ventricular tachycardia (VT) cycle length (CL) variability metrics could be used to discriminate between sustained and spontaneously terminating VT.

**Methods and results:**

In this single-centre retrospective study, we analysed data from 69 VT episodes stored on ICDs from 27 patients (36 spontaneously terminating VT, 33 sustained VT). Several VT CL parameters including heart rate variability metrics were calculated. Additionally, a first order auto-regression model was fitted using the first 10 CLs. Using features derived from the first 10 CLs, a random forest classifier was used to predict VT termination. Sustained VT episodes had more stable CLs. Using data from the first 10 CLs only, there was greater CL variability in the spontaneously terminating episodes (mean of standard deviation of first 10 CLs: 20.1 ± 8.9 vs. 11.5 ± 7.8 ms, *P* < 0.0001). The auto-regression coefficient was significantly greater in spontaneously terminating episodes (mean auto-regression coefficient 0.39 ± 0.32 vs. 0.14 ± 0.39, *P* < 0.005). A random forest classifier with six features yielded an accuracy of 0.77 (95% confidence interval 0.67 to 0.87) for prediction of VT termination.

**Conclusion:**

Ventricular tachycardia CL variability and instability are associated with spontaneously terminating VT and can be used to predict spontaneous VT termination. Given the harmful effects of unnecessary ICD shocks, this machine learning model could be incorporated into ICD algorithms to defer therapies for episodes of VT that are likely to self-terminate.

## Graphical Abstract

**Graphical Abstract:**
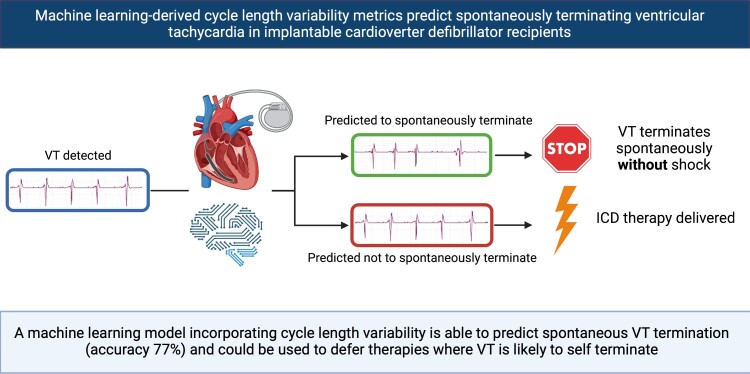

## Introduction

Implantable cardioverter defibrillators (ICDs) have been used for nearly 40 years in the management of patients at risk of ventricular arrhythmias (VA).^1^ Multiple randomized controlled trials have shown mortality reductions in both primary and secondary prevention settings.^2–6^

There has been a paradigm shift in ICD programming recently, with a move towards avoiding ICD shocks. Initially, rapid detection and treatment of ventricular tachycardia (VT) and ventricular fibrillation (VF) were emphasized; however more recently, there is mounting evidence that ICD shocks can be harmful and should be minimized when safe to do so.^7^ Implantable cardioverter defibrillator shocks reduce quality of life,^8^ may rarely be proarrhythmic,^9^ and have been associated with excess mortality.^10^ Potential mechanisms for harm include shock-induced myocardial injury or stunning.^11^

To reduce ICD shocks, current guidelines recommend longer detection times, allowing some episodes of VT to self-terminate without device therapy.^12^ The PREPARE, MADIT-RIT, RELEVANT, PROVIDE, and ADVANCE III studies^13–17^ have all shown the benefits of this approach. Once supraventricular tachycardia (SVT)/VT discrimination algorithms have diagnosed VT, detection and therapy are decided purely on VT cycle length (CL) and number of detection intervals.^18^

A potential additional parameter that can be used to identify self-terminating VT episodes is VT CL stability. Changes in VT CL stability over the duration of a VT episode have been described,^19^ and VT CL variation has been associated with spontaneous VT termination,^20^ though the evidence for this is derived predominantly from experiments or from the invasive electrophysiology laboratory setting. Supervised machine learning (ML) can be combined with biologically derived features to accurately predict outcomes. We used data from clinical VT episodes stored on ICDs to test our hypothesis that machine learning-derived VT CL stability features can be used to discriminate between sustained and spontaneously terminating VT, which may allow an ICD to defer therapy if a VT episode is predicted to be spontaneously terminating.

## Methods

This was a single-centre retrospective study, approved by the Health Research Authority (Integrated Research Application System ID: 293374, Research Ethics Committee reference: 21/PR/0108).

A review was conducted of the Boston Scientific Latitude Clinician Database to identify all adult patients with Boston Scientific ICDs for any indication, who were followed up at Imperial College Healthcare NHS Trust (London, UK). Four hundred thirty patients were identified. Each patient was reviewed for device-detected VT which self-resolved, required anti-tachycardic pacing (ATP) or required an ICD shock. Electrograms (EGMs) were thereafter examined, and episodes were included if a true VT episode lasting longer than 10 CLs was identified. To ensure reliable differentiation between VT and SVT, episodes where the atrial rate was equal to, or exceeded, ventricular rates and episodes from patients without atrial leads were excluded.

### Cycle length variability metrics

**Figure 1:**
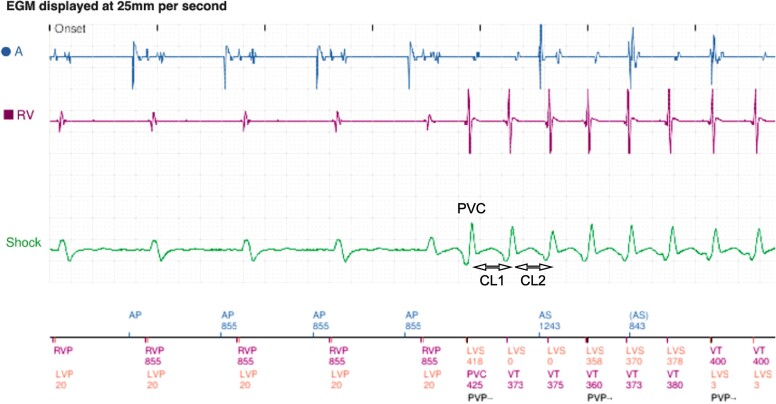
Cycle length measurement from device electrograms: example device electrograms obtained from hospital electronic records of Boston Scientific implantable cardioverter defibrillators. Premature ventricular contraction indicated the premature ventricular contraction that induces the ventricular tachycardia, CL1 indicates the first ventricular tachycardia cycle length, recorded as the difference in time (ms) between the premature ventricular contraction and the subsequent beat, which in this case this is 373 ms. Ventricular tachycardia cycle lengths for the subsequent beats were taken based on the device measurements (375, 360, 373 ms, etc.).

Device-measured consecutive CLs within each VT episode were collected. The first VT CL was taken as that following a premature ventricular contraction (PVC), and thus the first CL was recorded as the time between CL 1 of the VT and the preceding PVC (*Figure 1*). Where there was uncertainty about the initiation point of the VT, an electrophysiologist was consulted to adjudicate. All included episodes were subsequently divided into two groups: spontaneously terminating (those that self-terminated without therapy) or sustained (those that required ATP or shock). Patients with more than one VT episode could have VT episodes included in both sustained and spontaneously terminating groups of the study.

For each VT episode, several descriptive parameters were calculated, including mean of all CL, mean first CL, mean last CL, mean minimum CL, mean episode length, mean number of CLs, and mean standard deviation (SD) of the CLs. For the initiation-to-shortest CL analysis, three features were calculated: time taken to reach shortest CL (seconds), per cent of CL to reach shortest CL, and per cent change from first CL to shortest CL.

### Heart rate variability features

To assess the ability to predict sustained VT from stability parameters, several heart rate variability features were calculated. We used the differences between successive CLs to do this (inter-CL intervals). To assess the ability to predict spontaneous termination, we used features derived from the first 10 CLs, as previously described.^21^ These were as follows: mean, SD, root mean square of successive RR interval differences (RMSSDs), NN50, percentage of successive RR intervals that differ by more than 50 ms (pNN50), and the triangular interpolation of the NN interval histogram (TINN).

### Machine learning model

A first-order auto-regression model [AR(1) process] was fitted using the first 10 CLs. The AR coefficient, residual, and constant, together with other stability parameters, were then used as features for a random forest classifier to predict the probability of arrhythmia spontaneous termination. A random forest classifier is a type of machine learning algorithm that can combine multiple decisions trees to make predictions. Each tree is trained on a different subset features. Each tree independently makes predictions, and the final prediction of sustained vs. self-terminating VT is decided by majority voting.^22^ The features with an importance greater than the mean importance of all the features were selected for the final model. The selected features were evaluated using a k-fold cross validation, where k = 10.

### Statistical analysis

Student’s *t*-test was used for parametric data and Mann–Whitney *U* test for non-parametric data and considered statistically significant at *P* < 0.05. Analyses were performed using Matlab, Prism 8.0 software (GraphPad Software, CA, USA) and Python (version 3.9). Values are expressed as mean ± standard error or a median with interquartile range, for parametric and non-parametric data, respectively.

## Results

### Patient characteristics

Twenty-seven patients met the inclusion criteria, from which 69 VT episodes were included. There were 36 spontaneously terminating VT episodes from 19 patients and 33 sustained VT episodes from 12 patients. Four patients experienced both spontaneously terminating and sustained VT episodes. The median age was 66 (59–75), and 22 (81%) were male. Thirteen patients (48%) had a diagnosis of ischaemic cardiomyopathy. The indication for ICD implantation was for primary prevention in 13 (48%) patients. Further baseline characteristics in the two groups are displayed in *Table 1*. The median rate for the lowest therapy zone was 185 b.p.m. for the spontaneously terminating VT group and 160 b.p.m. for the sustained VT group.

**Table 1:** Baseline characteristics of patients grouped according to ventricular tachycardia outcome

	Spontaneously terminating	Sustained	*P*-value
**Number of patients**	19	12	
**Age**	62 (56–79)	66 (58–74)	0.98
**Male**	14 (73.7)	11 (91.7)	0.36
**Smoker**	2 (16.7)	1 (10)	0.99
**Left ventricular ejection fraction (%)**	36 (19–50)	43 (36–56)	0.18
**First therapy zone** **Heart rate (b.p.m.)**	185 (170–200)	160 (150–170)	<0.05
**Indication**			
** Primary**	11 (57.9)	3 (25.0)	0.14
** Secondary**	8 (42.1)	9 (75.0)	
**Type**			
** ICD**	10 (52.6)	10 (83.3)	0.13
** CRT-D**	9 (47.4)	2 (16.7)	
**Comorbidities**			
** IHD**	9 (50)	7 (54.3)	0.72
** Diabetes**	7 (43.8)	2 (27.3)	0.45
** AF**	6 (35.3)	2 (16.7)	0.41
**Disease group**			
** **ICM	8 (42.1)	7 (58.3)	
** **DCM	6 (31.6)	2 (16.7)	
** **Idiopathic VT	2 (10.5)	2 (16.7)	
** **HCM	1 (5.3)	0	
** **Sarcoid	1 (5.3)	0	
** **ACM	1 (5.3)	1 (8.3)	

ICD, implantable cardioverter defibrillator; CRT-D, cardiac resynchronization therapy defibrillator; IHD, ischaemic heart disease; AF, atrial fibrillation; ICM, ischaemic cardiomyopathy; DCM, dilated cardiomyopathy; VT, ventricular tachycardia; HCM, hypertrophic cardiomyopathy; ACM, arrhythmogenic cardiomyopathy.

### Sustained ventricular tachycardia had shorter first cycle length and shorter mean cycle length across the first 10 cycle lengths

The mean episode length and number of beats within each episode were longer in the sustained group (episode duration: sustained 11.2 ± 4.4 vs. spontaneously terminating 5.2 ± 1.9 s; number of beats: sustained 36 ± 14 vs. spontaneously terminating 16 ± 5 beats, *Table 2*, *P* < 0.0001). The mean first CL was shorter in the sustained group (329.9 ± 40 vs. 357.9 ± 41 ms, *P* < 0.05). The mean CL across the first 10 CLs was significantly lower in the sustained group (321.6 ± 64 vs. 344.1 ± 36 ms, *P* = 0.01). The mean CL across the episode trended towards being shorter in the sustained group but was not statistically significant.

**Table 2:** Mean ± standard deviation of ventricular tachycardia episode descriptors

	Spontaneously terminating	Sustained	*P*-value
Number of episodes (no.)	36	33	
Mean first CL (ms)	357.9 ± 41	329.8 ± 33	<0.05
Mean minimum CL (ms)	312.3 ± 33	305.7 ± 33	0.47
Mean episode duration (s)	5.2 ± 1.9	11.2 ± 4.4	<0.001
Mean number of beats (no.)	16 ± 5	36 ± 14	<0.001
Mean minimum CL across first 10 CLs	317.6 ± 32	306.7 ± 32	0.16
Mean CL across first 10 CLs	344.1 ± 36	321.6 ± 64	0.01
Mean 10th CL	337.4 ± 38	325 ± 38	0.18
Mean slowest tachycardia therapy zone detection time	7.1 ± 4.6	10.7 ± 3.0	<0.001

Sustained VT episodes had significantly lower mean first CL, mean CL across first 10 CLs, and longer mean episode length and number of beats.

CL, cycle length; VT, ventricular tachycardia.

### Sustained ventricular tachycardia episodes reach shortest cycle length faster than spontaneously terminating episodes

**Figure 2:**
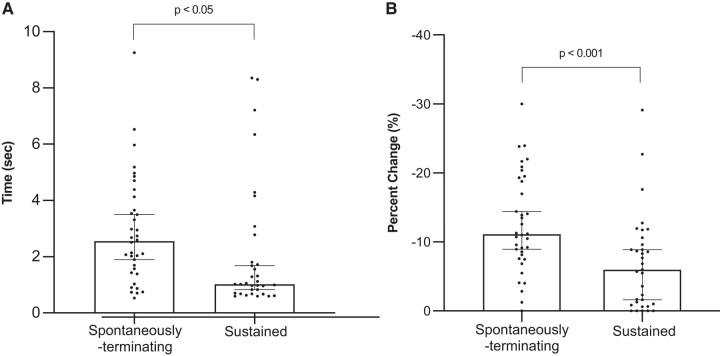
Initiation-to-shortest cycle length analysis: for sustained ventricular tachycardia, compared with spontaneously terminating ventricular tachycardia, there was a (*A*) shorter time to reach shortest cycle length (shortest ventricular tachycardia cycle length of the episode) and (*B*) reduced percentage change from first cycle length to shortest cycle length. Episodes of spontaneously terminating ventricular tachycardia *n* = 36, sustained ventricular tachycardia *n* = 33.

As shown in *Figure 2*, the sustained episodes reached their shortest CL within a median of 1.04 s (interquartile range 0.7–2.18 s) compared with 2.55 s (interquartile range 1.54–3.77 s) in the spontaneously terminating episodes (*P* < 0.05). Within the sustained episodes, the mean percentage decrease from initiation to minimum CL was significantly lower compared with spontaneously terminating episodes (6.0 ± 29% vs. 11 ± 30%; *P* < 0.001).

### Spontaneously terminating ventricular tachycardia episodes have more cycle length variability

**Figure 3:**
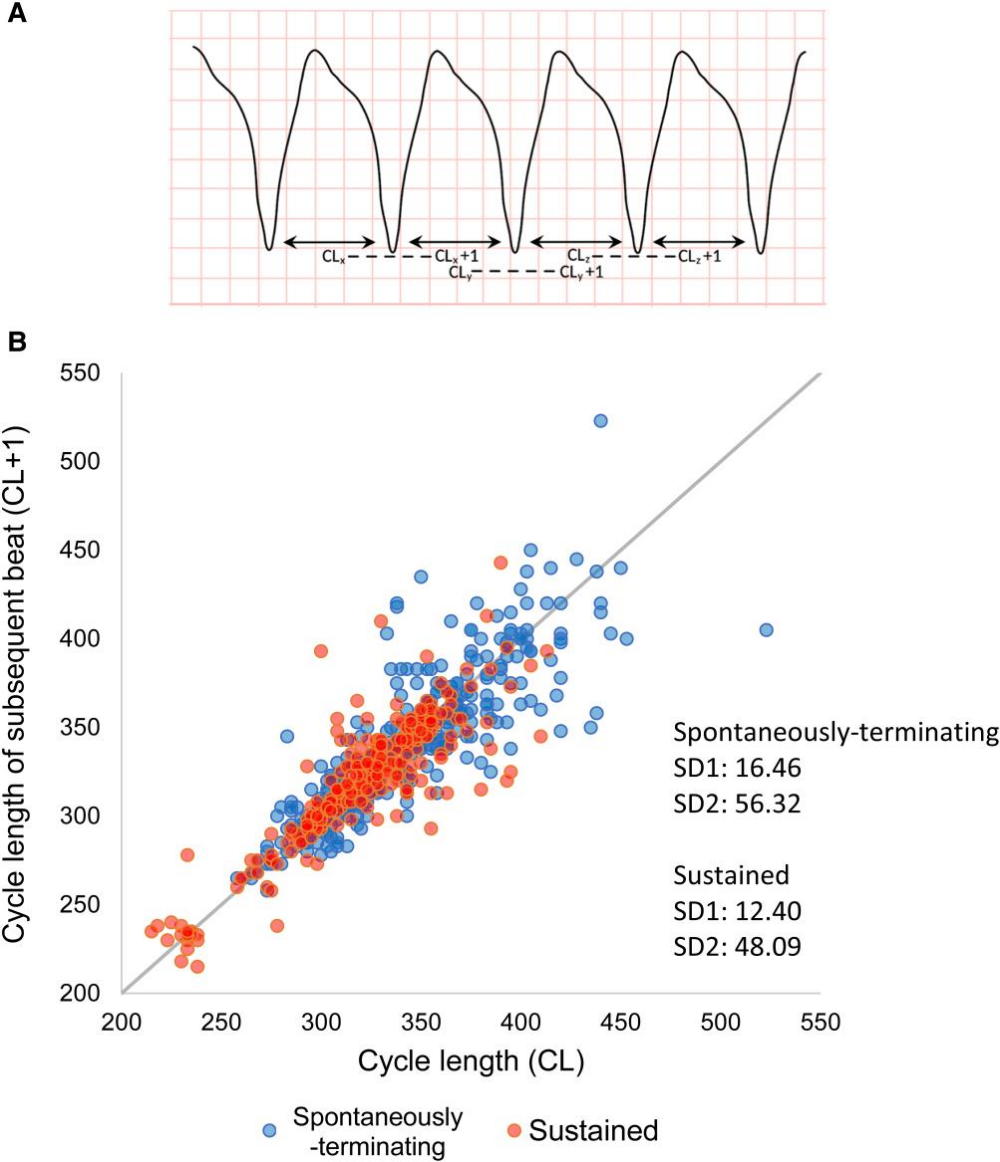
Spontaneously terminating ventricular tachycardia episodes showed increased beat-to-beat cycle length variation in the first 10 cycle lengths. (*A*) Schematic showing an episode of ventricular tachycardia. Each cycle length is paired (dashed line) with the subsequent cycle length (cycle length + 1). (*B*) A Poincaré plot of cycle length vs. cycle length + 1 for the first 10 cycle length s of each episode were plotted (total of 9 cycle length pairs per episode). This showed greater variability for the spontaneously terminating episodes (blue dots), with greater deviation from the grey line (cycle length = cycle length + 1). Episodes of spontaneously terminating ventricular tachycardia *n* = 36, sustained ventricular tachycardia *n* = 33. SD1, standard deviation of the Poincaré plot perpendicular to the line-of-identity; SD2, standard deviation of the Poincaré plot along the line-of-identity.

**Figure 4:**
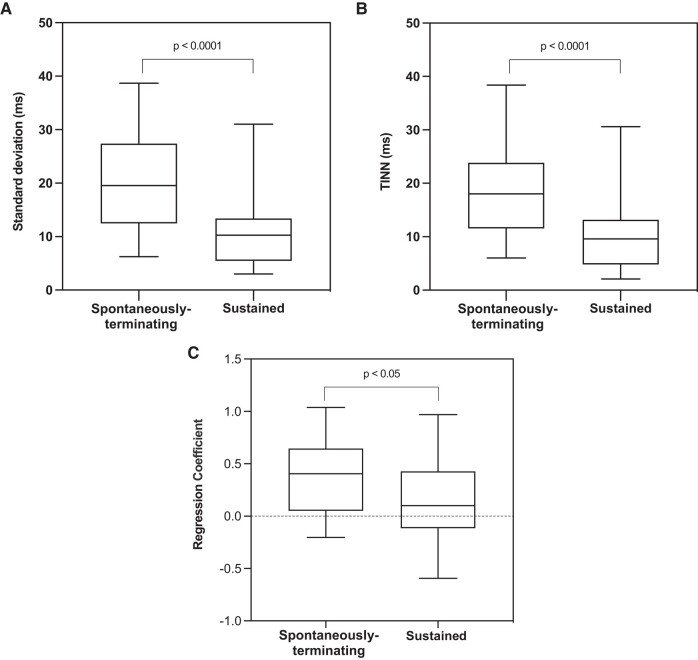
Spontaneously terminating ventricular tachycardia showed increased cycle length variation in the first 10 cycle lengths: spontaneously terminating ventricular tachycardia episodes had (*A*) higher standard deviation of successive interbeat intervals, (*B*) increased triangular interpolation of the NN interval histogram, and a (*C*) greater time series auto-regression coefficient. Episodes of spontaneously terminating ventricular tachycardia *n* = 36, sustained ventricular tachycardia *n* = 33.

**Figure 5:**
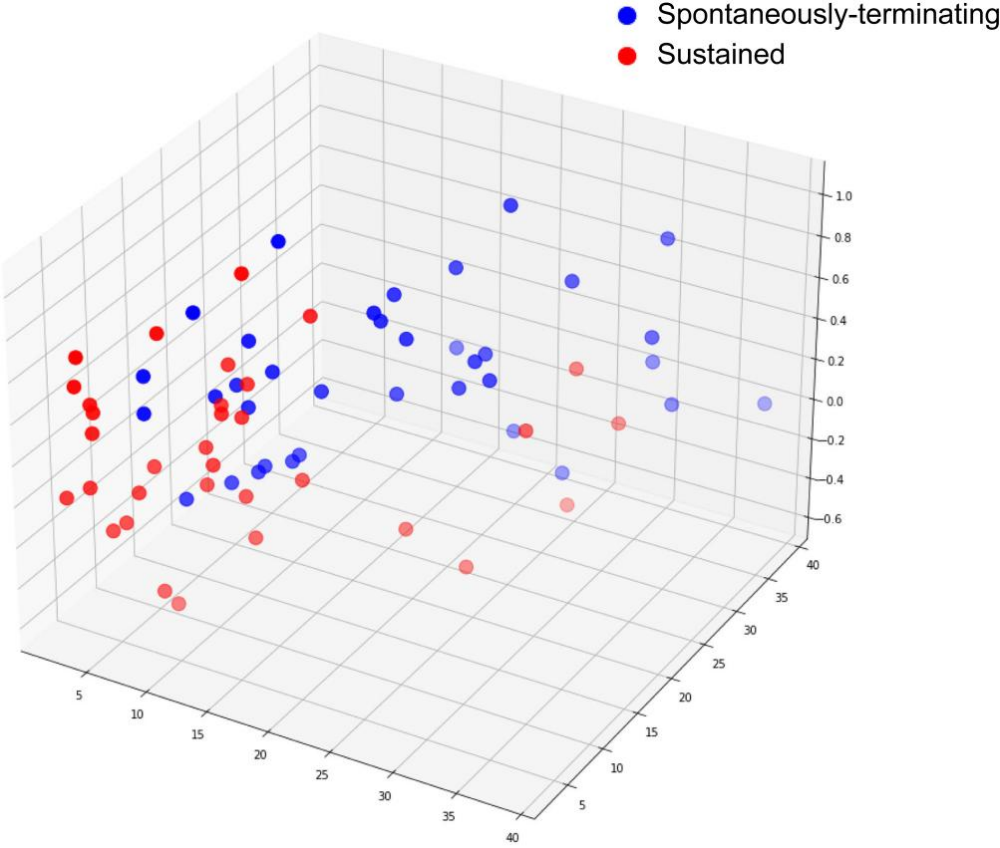
Three-dimensional scatter plot showing the feature distribution: cycle length standard deviation, auto-regression coefficient, and triangular interpolation of the NN interval histogram are plotted. There is separation of spontaneously terminating and sustained ventricular tachycardia cycle length features, with some overlap, suggesting that it is possible to use these features to distinguish spontaneously terminating ventricular tachycardia from sustained ventricular tachycardia episodes. Episodes of spontaneously terminating ventricular tachycardia *n* = 36, sustained ventricular tachycardia *n* = 33.

Across the entire episode, the mean of the standard deviation of successive CLs, a measure of CL variability within each episode, was greater in the spontaneously terminating episodes (21.3 ± 9.6 vs. 10.6 ± 5.3 ms; *P* < 0.0001). Including only the first 10 CLs of each VT episode, *Figure 3* shows the Poincaré plot of VT CL against the previous VT CL, showing the increased CL variability in the spontaneously terminating episodes. When considering just the first 10 CLs of each VT episode, several CL variability parameters were calculated. As shown in *Figure 4*, mean SD and TINN were greater in the spontaneously terminating episodes when compared with sustained VT episodes (mean SD 20.1 ± 8.9 vs. 11.5 ± 7.8 ms, *P* < 0.0001, mean TINN 18.6 ± 8.5 vs. 11.1 ± 8.0 ms, *P* < 0.001). Additionally, a first-order auto-regression model was fitted to each 10-CL series. The auto-regression coefficient was significantly greater in the spontaneously terminating episodes (mean auto-regression coefficient 0.39 ± 0.32 vs. 0.14 ± 0.39, *P* < 0.005, *Figure 4C*). *Figure 5* shows a 3D scatter plot of three features, with partial separation of spontaneously terminating and sustained VT data points.

### Machine learning-derived cycle length variability metrics can be used to predict whether ventricular tachycardia episode will be sustained

In order to determine whether outcome of the VT episode could be predicted using the first 10 CLs of each episode, the features above were fed into a random forest classifier, which is a form of supervised machine learning. The features used in the final model were mean CL, SD, RMSSD, TINN, AR coefficient, and constant. A k-fold cross-validation was performed, where k = 10. This resulted in an accuracy of 0.77 (95% confidence interval 0.67 to 0.87), a sensitivity of 0.79, a specificity of 0.75, a positive predictive value of 0.74, a negative predictive value of 0.79, and an area under the receiver operating characteristic of 0.80.

## Discussion

We report the first description of machine learning-derived VT CL variability features as a potential discriminator between sustained and spontaneously terminating ventricular arrhythmia, using data from clinical VT episodes recorded on ICD devices. Our data demonstrate the potential utility of CL variability in the first 10 CLs of VT to guide ICD therapies.

### Increased ventricular tachycardia cycle length stability is associated with sustained tachycardia requiring therapy

In this study, we found that sustained VT reaches shortest CL faster than spontaneously terminating episodes. Additionally, increased CL variability was associated with spontaneously terminating VT. In particular, the combination of 6 features in a random forest classifier, using only the first 10 CLs of the VT, was able to predict VT termination with an accuracy of 76%. Although the present accuracy is not sufficient to withhold therapies in all relevant cases, a proportion of VT episodes with the highest CL variability could have therapies delayed to reduce unnecessary shocks, where conventional programming might deliver a therapy, particularly at lower VT rates.

### Variability in cycle length occurs prior to tachycardia termination and is associated with spontaneously terminating tachycardias

Our findings are in agreement with previous studies that have associated CL variability with re-entrant tachycardia termination. Callans *et al.*^20^ studied 55 episodes of haemodynamically tolerated monomorphic VT during inpatient telemetry monitoring, ambulatory Holter monitoring, or electrophysiology studies, while Ortiz *et al.*^23^ utilized the canine sterile pericarditis model to study atrial flutter. Increased variability in CL was shown in both of these studies to occur just before termination. Similarly, studies on ibutilide have shown that it can terminate atrial flutter with increased CL variability before termination.^24^ Pogwizd *et al.*^25^ studied a group of six patients with prior myocardial infarction undergoing arrhythmia surgery and found that spontaneously terminating VT was associated with increased CL oscillations compared with sustained tachycardia and hypothesized that this may be due to dispersion of refractoriness.

Cismaru *et al.*^26^ describe a group of patients with VF in Brugada syndrome. They found self-terminating VF was associated with less CL variability than sustained VF requiring a shock. However, only five episodes of VF in this study were of spontaneous VF, and the remainder were during defibrillation threshold testing or programmed ventricular stimulation.

Our data are consistent with the above but are novel in that they are entirely derived from clinical VT episodes from patients with ICDs.

### Mechanism for association between cycle length variability and termination of re-entrant arrhythmias

In a canine atrial tissue model of atrial flutter, Frame and Simson^27^ and Frame and Rhee^28^ described that spontaneous CL oscillations were due to interval-dependent changes in conduction velocity action potential duration, which increase the likelihood of spontaneous tachycardia termination. Because of the restitution properties of myocardium, local changes in conduction occur due to the effects of the local CL and diastolic interval on the subsequent interval’s conduction velocity and action potential duration, respectively.^29^ These oscillations then increase the chances of the action potential wavefront encountering refractory tissue, leading to conduction block and tachycardia termination. Similar findings were subsequently described in a canine pericarditis model of atrial flutter, in the canine post-infarction heart,^23,30^ and in atrioventricular re-entrant tachycardia in humans.^31^

Although here we specifically discuss re-entrant arrhythmias, it is possible that some of our patients could have had triggered activity or automaticity driven VT. We could not differentiate mechanism in this study but were able to make the more clinically relevant distinction between sustained VT and spontaneously terminating VT.

### Machine learning for ventricular arrhythmia

Machine learning has been applied extensively in healthcare settings for risk prediction. In particular, ML has the potential to provide novel insights using the large amount of data collected by implantable cardiac devices. Machine learning has been recently successfully used to predict malignant ventricular arrhythmias in patients with implantable cardiac devices.^32^ A multivariate approach using cardiac imaging has also been successfully used to predict sudden cardiac death.^33^ Machine learning models using ICD data can also be used to predict electrical storm.^34^ Distinct to the previous work, we describe for the first time a ML model that can predict the outcome of a VT event, with the potential to prevent unnecessary and harmful ICD shocks.

### Avoiding harmful implantable cardioverter defibrillator shocks

Observational data suggest that appropriate ICD shocks are associated with increased mortality.^35^ In particular, the finding that ATP is not associated with increased mortality reaffirms the potential harmful effects of ICD shocks beyond the effects of VT itself.^35^ Implantable cardioverter defibrillator shocks reduce quality of life,^8^ may rarely be proarrhythmic,^9^ and have been associated with excess mortality.^10^ Potential mechanisms for increased mortality include shock-induced myocardial injury or stunning.^11,36^ There is therefore strong evidence for the avoidance of ICD shocks, unless absolutely necessary.

Historically, there has been a focus on avoiding ‘inappropriate’ therapies, e.g. due to SVT rather than a ventricular arrhythmia. However, more recently multiple randomized controlled trials have demonstrated the benefits of more conservative programming to reduce ‘unnecessary’ ICD therapies for VT that are either not haemodynamically compromising or may self-terminate.^13–17^ Germano *et al.*^37^ showed that ICD shocks in the major ICD randomized controlled trials out-numbered sudden cardiac deaths in the control group by a factor of 2–3. While a proportion of these are inappropriate shocks, even appropriate shocks out-number sudden cardiac deaths in the control groups, indicating that a proportion of these shocks are potentially ‘unnecessary’ as the VT would not have been haemodynamically significant, would have self-terminated, or both. Methods to accurately identify the episodes that are likely to self-terminate are therefore sorely needed.

### Cycle length variability could be used to delay therapies for ventricular tachycardia that are likely to be spontaneously terminating

Currently, CL stability is often used as an SVT discriminator to reduce inappropriate shocks that may occur due to AF with a rapid ventricular response.^18^ Our data suggest that the CL stability or variability may be used to delay therapies for VT episodes that may spontaneously terminate and to prevent unnecessary shocks. Incorporating this feature in ICD detection algorithms has the potential to further reduce unnecessary shocks for VTs that will self-terminate. In particular, this could be incorporated into the first VT zone where tachycardia rate is likely to be lower and therefore more likely to be tolerated haemodynamically by the patient. Cycle length variability applied to first 10 CLs (lasting ∼3.4 s) would allow ample time to defer a therapy if appropriate. Additionally, CL variability could be combined with quantification of electromechanical coupling to identify episodes most suited for delaying therapy.^38^ In order to prevent incorrectly withholding therapies for polymorphic VT and VF, an upper rate boundary would be incorporated, above which CL variability would not be used to withhold therapies.

It should be noted that the spontaneously terminating VT episodes in this study did not receive therapies even with existing programming algorithms and settings so would not have benefited from additional algorithms to delay therapy. However, we postulate that a subset of episodes in our sustained group that did receive therapies, with the highest CL variability, may have self-terminated if CL variability was incorporated as an additional feature and therapy delayed, though this needs to be tested prospectively. Similarly, longer episodes that spontaneously terminate may be treated under current programming guidelines, which could be avoided with a CL variability algorithm.

Finally, an alternative approach would be to programme very long detection/therapy times and use CL variability to identify episodes of VT that are highly likely to be sustained. Anti-tachycardic pacing in particular could then be delivered early in order to minimize symptoms and potentially prevent syncope. This would need to be balanced against a risk of VT acceleration and need for an ICD shock.

### Limitations

Our findings should be regarded as hypothesis generating. Given the relatively small dataset, further work is needed to validate our findings against both an internal and external dataset, and subsequently prospective evaluation is needed. In particular, further data from patients with a wide-spectrum of cardiac disease are needed. Given the small number of patients, we were unable to assess the performance of the algorithm when spontaneously terminating and sustained episodes occurred in the same patient. We did not distinguish between monomorphic and polymorphic VT in the present study; as discussed above, this would be important in future studies to mitigate the risk of untreated polymorphic VT. More data would likely allow improved accuracy in prediction of VT termination using CL variability and may allow the use of deep learning in addition to or instead of hand-crafted feature extraction. The therapy zones were on average programmed at a lower heart rate threshold in the sustained group. If anything, this may have diluted the differences seen between groups in our CL variability analysis, as some events that would have self-terminated may have received therapy delivered due to the more aggressive programming. Only ICDs from a single manufacturer were included in this study, although it is unlikely that CL variability is dependent on device manufacturer; further work is needed to expand the dataset to include all major device manufacturers. It has been reported that anti-arrhythmics can affect CL.^39^ Unfortunately, we were unable to obtain contemporary medication lists at the time of the VT events.

## Conclusion

Using data from clinical VT episodes recorded on ICD devices, we describe that increased VT CL variability is associated with self-terminating spontaneously terminating VT and can be used to predict spontaneous VT termination. Given the harmful effects of unnecessary ICD shocks, this ML model could be incorporated into ICD algorithms to defer therapies for episodes of VT that are likely to self-terminate.

## Data Availability

The data analysed in this study are not publicly available due to ethical restrictions.
